# Idiopathic adult intussusception

**DOI:** 10.1186/1865-1380-4-8

**Published:** 2011-03-16

**Authors:** Sanooj Soni, Philip Moss, Thiagarajan Jaiganesh

**Affiliations:** 1 St Georges Hospital, Blackshaw road, Tooting, London, SW17 0QT, UK

## Abstract

Intussusception is an uncommon cause of abdominal pain in adults and poses diagnostic challenges for emergency physicians, due to its varied presenting symptoms and time course. Diagnosis is thus often delayed and results in surgical intervention due to the development of bowel ischaemia. We report on a young patient who presented with an ileo-ileal intussusception in whom there were no underlying lesions identified as a causal factor.

## Case Report

**Figure 1:**
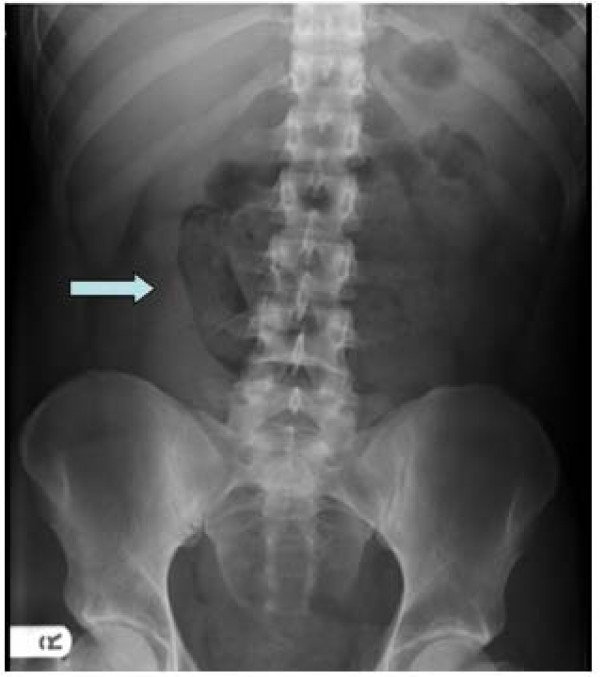
**Plain abdominal x-ray showed a single loop of dilated small bowel (*arrow key*)**.

A 26-year-old male, with no prior medical history, presented to the emergency department with a 24-h history of bouts of severe colicky abdominal pain, worse in the left lower quadrant. The symptom had initially started with vomiting just prior to the abdominal pain. He subsequently developed some diarrhoea, further episodes of vomiting and began to feel unwell with a fever. He was unable to tolerate even oral fluids, which had prompted his presentation to the ED that morning. There was no episode of rectal bleeding. His temperature was 38°C, pulse of 120 beats per minute and a respiratory rate of 28 breaths per minute. He remained normotensive and maintained good oxygen saturations. Examination revealed a soft abdomen but gross tenderness in the lower quadrants, worse in the left iliac fossa. There was no palpable mass, and rectal examination did not demonstrate any blood. Bowel sounds were present and there were no clinical signs of peritonitis. After blood investigations were sent, he was treated with intravenous paracetamol, hyoscine butylbromide and intravenous fluids. He was sent for an abdominal x-ray, which revealed a single dilated loop of small bowel (3 cm) in the central abdomen with scanty bowel gas elsewhere (Figure 1). He subsequently was given opioid analgesia as his pain was increasing in severity.

**Figure 2:**
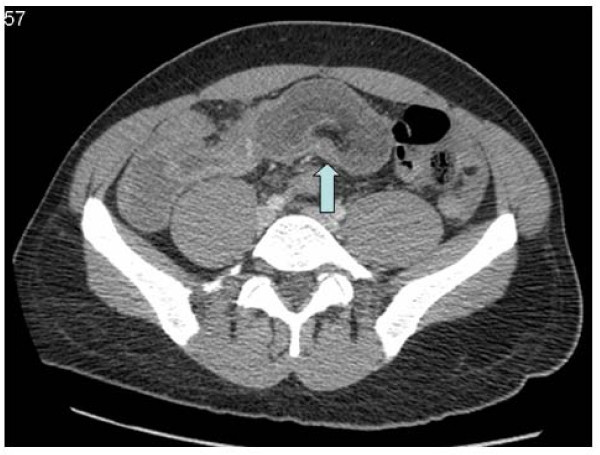
**A sausage-shaped mass (*arrow key*) represents the intussuscepted segment**. The fat density seen in the centre represents mesenteric fat.

**Figure 3:**
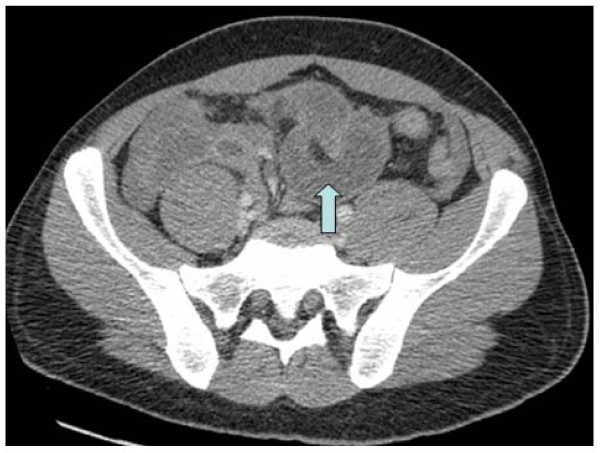
**CT scan depicted the origin (*arrow key*) of the telescoping of the ileal segment**.

An arterial blood gas on air analysis revealed a respiratory alkalosis (pH 7.650, pCO_2 _2.33 kPa, pO2 14.0 kPa, base excess 1.5 mmol/l and bicarbonate 25.6 mmol/l). He had a raised lactate level of 3.5 mmol/l. Other blood tests illustrated raised acute inflammatory markers such as C-reactive protein of 231.7 ng/ml, and a white cell count of 15.9 × 10^9^/l with a neutrophil count of 13.7 × 10^9^/l. Given his extreme pain, fever and raised lactate level, a clinical diagnosis of intra-abdominal sepsis secondary to gut ischaemia was made and the patient referred to the surgical team. A preoperative CT scan of his abdomen revealed an ileo-ileal intussusception with several loops of dilated small bowel proximal to the intussusception (Figures 2 and 3). There was also a large amount of free fluid seen in the abdomen.

He underwent a laparotomy a few hours after his presentation to the ED. Three litres of serosanguinous fluid was found in his peritoneal cavity along with 20 cm of ischaemic small bowel. This portion of the small bowel was resected (29 cm about 15 cm from the ileocaecal valve) and a primary end-to-end anastomosis was performed. He was transferred to the intensive care unit postoperatively for optimisation. He made a good recovery and was discharged from the hospital 5 days later. Histopathology results of the removed specimen confirmed an intussuscepted segment of small bowel, which demonstrated a spectrum of changes from mucosal ischaemia/infarction to transmural haemorrohagic infarction. There was no evidence of malignancy or any other pathological trigger/nidus, and therefore the aetiology of his intussusception was unknown.

## Discussion

Abdominal pain, which comprises about 5 to 10 percent of emergency department (ED) visits, continues to pose diagnostic challenges for emergency physicians because of the wide range of differential diagnoses, including gastrointestinal, gynaecological, genitourinary and cardiopulmonary causes [1]. Adult intussusceptions poses a further challenge as they often present with nonspecific symptoms and run a chronic indolent course until bowel ischaemia supervenes [2].

Intussusceptions occur when one segment of the gastrointestinal tract (intussusceptum) telescopes into the lumen of an adjacent distal segment of the gastrointestinal tract (intussuscipiens). Adult intussusceptions represent only about 5% of all intussusceptions [3] and thus a rare cause of hospital admissions, accounting for only 0.005% [4].

Intussusception remains a rare clinical entity in adults. The mean age is 54.4 years, and the male-to-female ratio is 1:1.3 [5]. In adults, cases can be either acute or chronic, and abdominal pain is the most common symptom (71-100%), followed by nausea and vomiting in 40-60% of the cases. Bleeding per rectum was seen in 4-33% of the cases [6]. This wide range is usually based on the site of the intussusception, with colonic ones bleeding more frequently than the ileal varieties. Acute abdominal pain with guarding is present in only about 50% of the cases [7]. Abdominal masses are palpable in less than 10% of patients [8].

A classification system exists according to the location of the intussusception. The four types are ileo-colic, ileo-ileo-colic, colo-colic and small bowel intussusception (jejuno-jejunal and ileo-ileal) [9]. In adults, often there is an underlying trigger or nidus for the intussusception in around 90-95% of the cases [10]. The majority of lead points in the small intestine consist of benign lesions, such as benign neoplasms, Meckel's diverticuli, appendix and adhesions. Twenty-five percent of small bowel intussusceptions are caused by malignant lesions, whereas in the large bowel this number increases to around 50% [11].

Abdominal CT is the most useful diagnostic tool not only for detecting an intussusception with a diagnostic yield of around 78%, but also helps in identifying the underlying cause [12]. The CT appearance of an intussusception is often a complex sausage-shaped soft tissue mass with an eccentric area of fat density contained within, which represents the mesenteric fat. The mesenteric vessels may be visible [13]. Plain abdominal x-rays and ultrasound are of limited diagnostic value in adults.

Treatment is almost always surgical in adults when compared to children and invariably leads to resection of the involved bowel segment with subsequent primary anastomosis. Gastroduodenal and coloanal intussusceptions are extremely rare and may require innovative surgical techniques [14]. Intermittent intussusceptions are known to occur and are often seen in either barium follow-through studies or on CT scans in patients with celiac disease, Crohn's disease, intestinal tumours and malabsorption syndromes as a result of abnormal intestinal contractions [15]. These transient ones can be managed conservatively in the absence of any severe abdominal symptoms.

Although, intussusceptions themselves have a good prognosis, it is often the nature of the lesion causing the intussusception on which the decisive factor is expected. Mortality for adult intussusceptions increases from 8.7% for the benign lesions to 52.4% for the malignant variety [8]. In our case, no clear nidus or trigger was identified on histological examination of the resected segment.

## Conclusion

Adult intussusception is a rare but well-recognized condition. A high index of suspicion and early diagnosis with a CT scan will identify patients requiring emergent surgery and thus prevent serious complications such as haemorrhage, intestinal gangrene and perforation.

## Consent

Consent was obtained from the patient for publication of this case report and accompanying images.

## Competing interests

The authors declare that they have no competing interests.

## Authors' contributions

SS - Wrote the first draft of the paper and coordinated the review of all the drafts. PM - Reviewed all drafts of the paper. TJ - Reviewed and commented on all the drafts of the paper and on all radiographic images. All authors read and approved the final manuscript.
